# Age discrimination and sleep quality in older adults: Depression as mediator

**DOI:** 10.1093/geroni/igaf122.2197

**Published:** 2025-12-31

**Authors:** YoungBin Koh, Yeonjung Lee

**Affiliations:** Chung-Ang University, Seoul, Republic of Korea; Chung-Ang University, Seoul, Republic of Korea

## Abstract

Though Korean society has traditionally emphasized respect for older adults, these values are fading in contemporary times, resulting in widespread age discrimination and hostility toward older adults. Stress process model suggests that life events such as discriminatory experience is a substantial stressor and contributes to chronic strain, which, in turn, those have been linked to a number of adverse outcomes in older adults. Previous studies suggest that perceived age discrimination adversely affects older adults’ mental, physical, and social health. Additionally, there is a growing interest that discrimination may be a contextual contributor to sleep problems in later life. However, little attention has been paid to the stress process of age discrimination, particularly whether depression mediates the relationship between perceived age discrimination and sleep problems. The purpose of the study is to examine the pathway of how age discrimination contributes to depression, ultimately affecting sleep quality. Using data from the 2023 Senior Citizen Survey (N = 9,818), we applied PROCESS Macro Model 4 to assess the mediating role of depression. Results show that age discrimination is significantly associated with increased depression, which, in turn, negatively impacts sleep quality. The results suggest that the effects of age discrimination on sleep operate indirectly through heightened depression. These findings have implications for policy and practice, emphasizing the need for strengthening the laws to prevent age discrimination and expanding depression management programs to raise awareness of the consequences of age discrimination.

